# Genomic instability-derived plasma extracellular vesicle-microRNA signature as a minimally invasive predictor of risk and unfavorable prognosis in breast cancer

**DOI:** 10.1186/s12951-020-00767-3

**Published:** 2021-01-12

**Authors:** Siqi Bao, Ting Hu, Jiaqi Liu, Jianzhong Su, Jie Sun, Yue Ming, Jiaxin Li, Nan Wu, Hongyan Chen, Meng Zhou

**Affiliations:** 1 School of Biomedical Engineering, School of Ophthalmology & Optometry and Eye Hospital, Wenzhou Medical University, Wenzhou, 325027 People’s Republic of China; 2 State Key Laboratory of Molecular Oncology, National Cancer Center/National Clinical Research Center for Cancer/Cancer Hospital, Chinese Academy of Medical Sciences and Peking Union Medical College, Beijing, 100021 People’s Republic of China; 3 Department of Breast Surgical Oncology, National Cancer Center/National Clinical Research Center for Cancer/Cancer Hospital, Chinese Academy of Medical Sciences and Peking Union Medical College, Beijing, 100021 People’s Republic of China; 4 PET-CT Center, National Cancer Center/Cancer Hospital, Chinese Academy of Medical Sciences and Peking Union Medical College, Beijing, 100021 People’s Republic of China; 5 Department of Orthopedic Surgery, Beijing Key Laboratory for Genetic Research of Skeletal Deformity & Key Laboratory of Big Data for Spinal Deformities, State Key Laboratory of Complex Severe and Rare Diseases, All at Peking Union Medical College Hospital, Peking Union Medical College and Chinese Academy of Medical Sciences, Beijing, 100730 People’s Republic of China

**Keywords:** Breast cancer, Genomic instability, Extracellular vesicle, Exosomes, microRNA

## Abstract

**Background:**

Breast cancer (BC) is the most frequently diagnosed cancer and the leading cause of cancer-associated deaths in women. Recent studies have indicated that microRNA (miRNA) regulation in genomic instability (GI) is associated with disease risk and clinical outcome. Herein, we aimed to identify the GI-derived miRNA signature in extracellular vesicles (EVs) as a minimally invasive biomarker for early diagnosis and prognostic risk stratification.

**Experimental design:**

Integrative analysis of miRNA expression and somatic mutation profiles was performed to identify GI-associated miRNAs. Then, we constructed a discovery and validation study with multicenter prospective cohorts. The GI-derived miRNA signature (miGISig) was developed in the TCGA discovery cohort (n = 261), and was subsequently independently validated in internal TCGA validation (n = 261) and GSE22220 (n = 210) cohorts for prognosis prediction, and in GSE73002 (n = 3966), GSE41922 (n = 54), and in-house clinical exosome (n = 30) cohorts for diagnostic performance.

**Results:**

We identified a GI-derived three miRNA signature (*MIR421*, *MIR128-1* and *MIR128-2*) in the serum extracellular vesicles of BC patients, which was significantly associated with poor prognosis in all the cohorts tested and remained as an independent prognostic factor using multivariate analyses. When integrated with the clinical characteristics, the composite miRNA-clinical prognostic indicator showed improved prognostic performance. The miGISig also showed high accuracy in differentiating BC from healthy controls with the area under the receiver operating characteristics curve (ROC) with 0.915, 0.794 and 0.772 in GSE73002, GSE41922 and TCGA cohorts, respectively. Furthermore, circulating EVs from BC patients in the in-house cohort harbored elevated levels of miGISig, with effective diagnostic accuracy.

**Conclusions:**

We report a novel GI-derived three miRNA signature in EVs, as an excellent minimally invasive biomarker for the early diagnosis and unfavorable prognosis in BC.

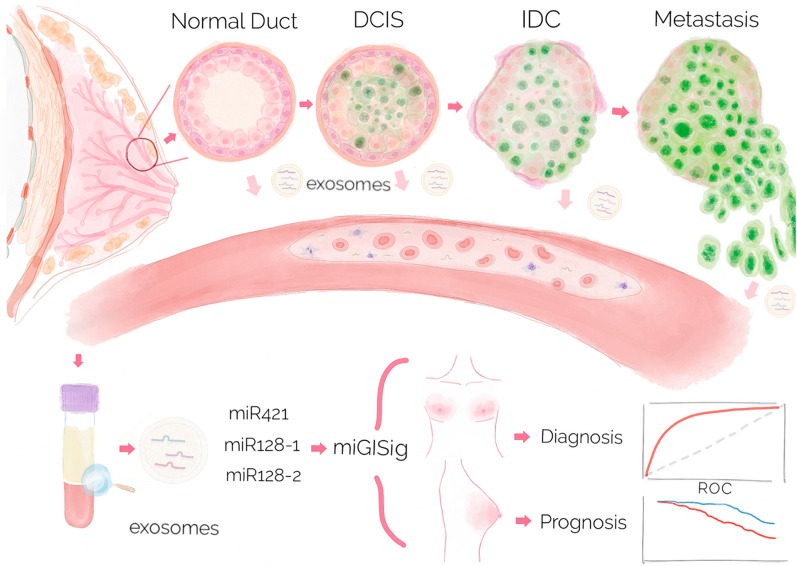

## Background

Breast cancer (BC) is one of the most commonly diagnosed cancer types, accounting for 30% of all new cancer diagnoses in women. The incidence rate of BC has remained generally stable over the past few decades [1]. Despite the recent improvements in detection methods and therapeutic choice, BC remains a significant public health issue worldwide, and the clinical outcome of patients varies markedly due to the stage at diagnosis and molecular subtype [2]. Classic clinicopathological features, including tumor size, histological subtypes and grades, lymphatic invasion, lymph node metastasis, estrogen receptor (ER), progesterone receptor (PR), and human epidermal growth factor receptor 2 (HER2) have effective value in guiding clinical decisions; Serum tumor markers, such as carcinoembryonic antigen (CEA), cancer antigen 19-9 (CA19-9), cancer antigen 125 (CA125), cancer antigen 15-3 (CA15-3), have been typically used for follow-up monitoring [3, 4]. However, BC patients are still faced with variable biological and clinical behaviors, due to high molecular and cellular heterogeneity [5, 6]. Therefore, it is urgently needed to identify reliable and robust biomarkers to enhance early risk detection and prognosis prediction with minimal invasion for improving clinical management and treatment decision-making for BC patients.

Genomic instability (GI) is defined as a process in which genomic changes are prone to increase and can influence the phenotype [7, 8], and has been recognized as an evolving hallmark or characteristic of most types of cancer [9]. GI promotes inter- and intra-tumor heterogeneity and is a major driving force for cancer cells to survive, proliferate, and disseminate [10]. Frequent GI was commonly observed in BC cells, including numerical and structural genomic changes [11, 12]. The molecular basis of GI in BC has not been fully elucidated, and BC can be characterized by vast GI-derived heterogeneity; therefore, understanding the molecular basis of GI in BC would not only enable the molecular etiology and pathobiology of BC to be determined, but also to develop improved cancer prevention, diagnosis, and prognosis methods [13, 14].

MicroRNA (miRNA) is a type of small, evolutionarily conserved, single-stranded, non-coding RNA, that induces mRNA degradation or translational repression by completely or incompletely binding to the target mRNA [15–17]. Accumulating evidence has highlighted the important roles of miRNAs, as crucial master regulators of various biological processes, including cell differentiation, development, and homeostasis [18, 19]. Deregulation of miRNA function plays a vital role in cancer pathogenesis [20–23]. Previous studies have revealed that several miRNAs are involved in enhancing GI by impairing DNA repair or preventing GI by enhancing the response to DNA damage [24]. In addition, GI may be mediated by horizontal transfer of tumor-derived macromolecules, such as miRNAs, via EVs [25]. Whether these alterations of GI-associated miRNAs can be detected in EVs, and therefore have clinical utility as promising minimally invasive biomarkers in BC, however, has not been investigated.

In this study, we aimed to identify miRNAs involved in GI based on the mutator hypothesis by integrating expression and somatic mutation profiles, and develop a GI-derived miRNA signature (miGISig) for early diagnosis and prognosis prediction of BC patients. In addition, we also uncovered the functional roles of the newly identified three miRNAs in the regulation of GI and finally, we assessed the use of the miGISig, as a minimally invasive biomarker in circulating exosomes to identify BC patients from asymptomatic controls.

## Results

### Identification of GI-associated miRNAs in BC

**Fig. 1:**
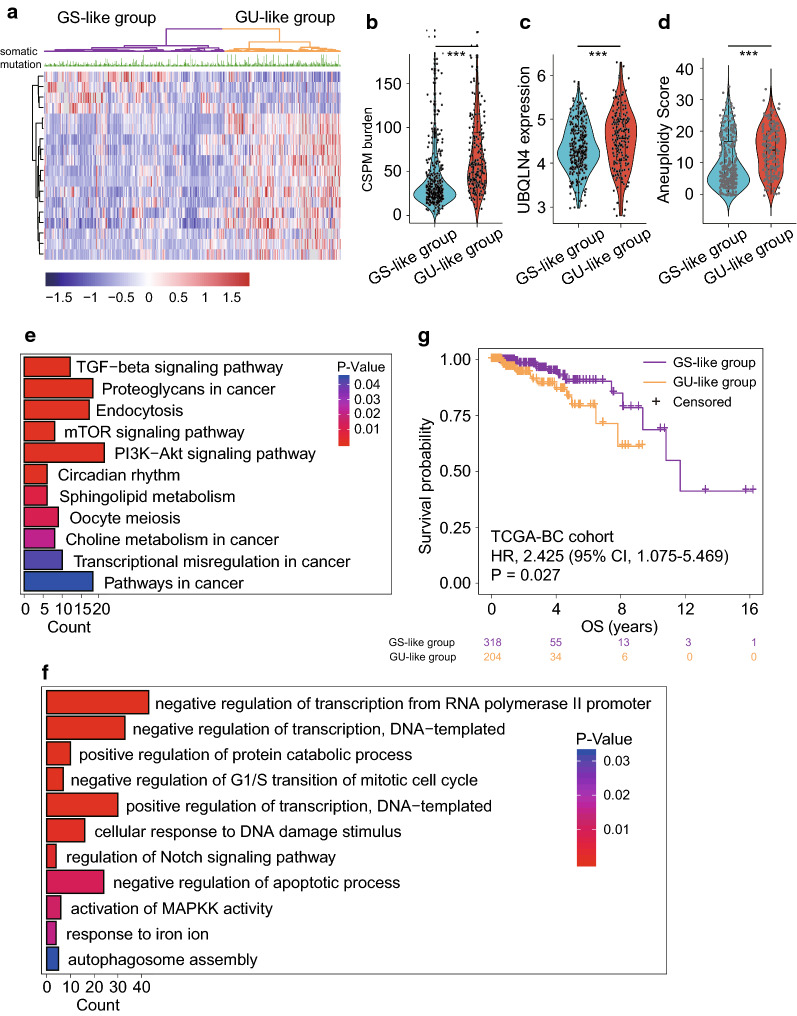
Identification and functional characterization of GI-associated miRNAs in BC patients. **a** Unsupervised clustering of 522 BC patients based on the expression pattern of 18 DEmiRNAs. The left purple cluster is GS-like group, and the right khaki cluster is GU-like group. Violin diagram of CSPM burden (**b**), UBQLN4 expression level (**c**) and aneuploidy scores (**d**) in the GU-like group and GS-like group. Horizontal lines: median values. Statistical analysis was performed using the Mann–Whitney U test. *P-value < 0.05, **P-value < 0.01, ***P-value < 0.001. **e**, **f**. Functional enrichment analysis of KEGG and GO for target genes of miRNA. G. Kaplan–Meier estimates of overall survival of patients in the GU-like group and GS-like group. HRs and 95% CIs for high vs. low miGISig score was estimated using the univariate Cox analysis. P values comparing risk groups were calculated with the log-rank test. *BC* breast cancer, *CIs* confidence intervals, *GI* Genome instability, *CSPM* cumulative somatic point mutation, *GO* Gene Ontology, *GS* genomically stable, *GU* genomically unstable, *HRs* Hazard ratios, *KEGG* Kyoto Encyclopedia of Genes and Genomes

Differential analysis of miRNA expression was performed between the GU-like and GS-like groups using TCGA-BC cohort, in which, 18 differentially expressed miRNAs (DEmiRNAs) were identified (Additional file 1: Table S4). Among them, 14 and 4 miRNAs were found to be up- and downregulated, respectively, in the GS-like tumors compared with that in the GU-like tumors. The unsupervised hierarchical clustering analysis based on the expression level of the 18 DEmiRNAs produced two patient clusters: a GS-like cluster (n = 318) and a GU-like cluster (n = 204), respectively (Fig. 1a). There was a significantly high frequency of somatic mutations in the GU-like group compared with that in the GS-like group (median value 57.5 vs. 29; P < 0.001, Mann–Whitney U test; Fig. 1b). Meanwhile, the expression level of the *UBQLN4* gene and aneuploidy score in the GU-like group was significantly higher compared with that in the GS-like group (P < 0.001, Mann–Whitney U test; Figs. 1c and 1d). Functional enrichment analysis of the 18 DEmiRNAs-specific mRNA targets identified several enriched biological processes and pathways (Figs. 1e and 1f), including transforming growth factor (TGF)-β, regulation of transcription, negative regulation of G_1_/S transition of mitotic cell cycle and cellular response to DNA damage stimulus, and the Notch signaling pathway, which are all known to be GI-related biological pathways. Furthermore, there was a significant difference in OS time between the GU-like and the GS-like groups (median OS, 11.7 vs. > 16 years; P = 0.027, log-rank test; Fig. 1g). These results suggested that the 18 DEmiRNAs were involved in GI and associated with BC patients' prognosis.

**Fig. 2:**
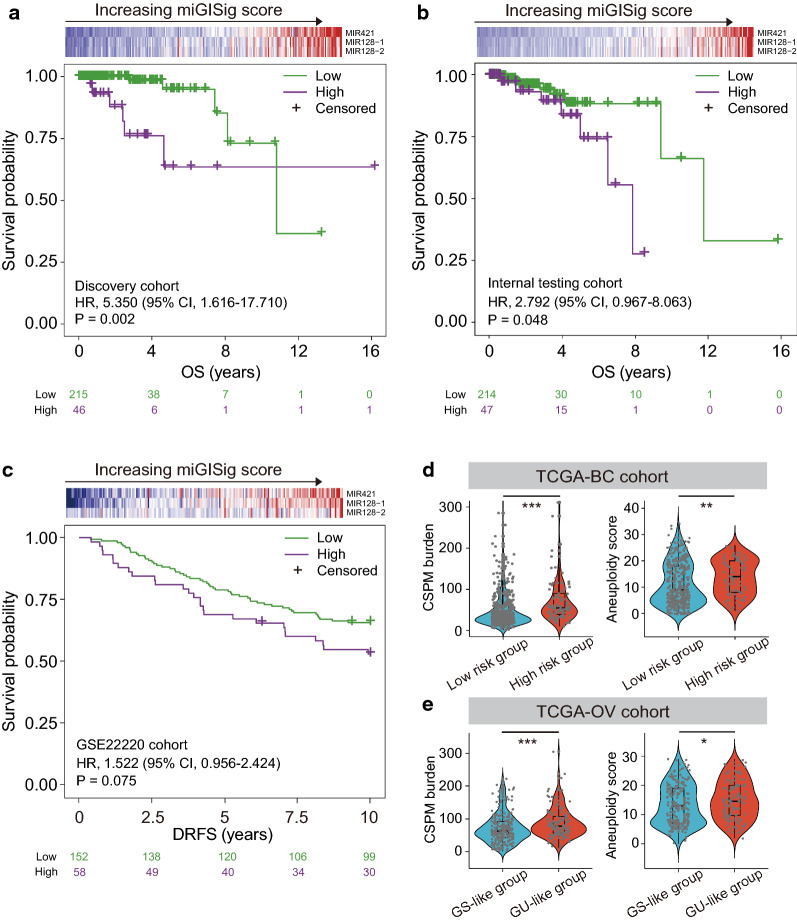
Development and validation of the miGISig in prognostic risk stratification. Kaplan–Meier estimates of OS or DRFS of patients with low or high miGISig score in the discovery cohort (**a**), internal testing cohort (**b**) and GSE22220 cohort (**c**). HRs and 95% CIs for high vs. low miGISig score were estimated using the univariate Cox analysis. P values comparing risk groups were calculated with the log-rank test. Violin diagram of CSPM burden and aneuploidy scores in the low risk group and high risk group in TCGA-BC cohort (**d**) and in the GS-like group and GU-like group in TCGA-OV cohort (**e**). Statistical analysis was performed using the Mann–Whitney U test. *P-value < 0.05, **P-value < 0.01, ***P-value < 0.001. *BC* breast cancer, *CIs* confidence intervals, *CSPM* cumulative somatic point mutation, *DRFS* Distant relapse-free survival, *GI* Genome instability, *GS* genomically stable, *GU* genomically unstable, *HRs* Hazard ratios, *OS* overall survival

### Development and validation of a GI-derived miRNA signature for prognostic risk stratification

We first analyzed the panel of 18 GI-related miRNAs using the univariate and multivariate Cox proportional hazard regression analysis in the TCGA discovery cohort and identified three-miRNA signature (hereafter referred to as miGISig) as follows: miGISig score = (0.501 × expression level of *MIR421*) + (0.6808 × expression level of *MIR128-1*) + (− 0.3617 × expression level of *MIR128-2*) (Additional file 1: Table S5). When stratified by the optimal cut-off value (cut-off, 0.516), the miGISig demonstrated significant prognostic value, as shown by the Kaplan–Meier curves of the risk groups, with high miGISig demonstrating poor prognosis (HR = 5.350, 95% CI 1.616–17.710; P = 0.002, log-rank test; Fig. 2a). The 5-year OS rates for miGISig-driven low-risk and high-risk groups were 94.8 and 63.5%, respectively. The prognostic value of the miGISig was subsequently validated in multiple independent datasets, which were in agreement with the findings driven from the initial TCGA discovery cohort. The miGISig achieved significant or marginally significant discrimination for the survival time (internal testing cohort, P = 0.048, and GSE22220 cohort, P = 0.075, log-rank test; Fig. 2b, c). These results demonstrated the robust performance of the miGISig in predicting unfavorable prognosis in BC patients.

Next, we further examined the relationship between miGISig and CSPM burden, and aneuploidy score. In the TCGA-BC cohort, the CSPM burden and aneuploidy score of patients in the high-risk group was significantly higher compared with that in patients in the low risk-group (median somatic mutations 56 vs. 34, P < 0.001; and median aneuploidy score 14 vs. 9, P = 0.002; Mann–Whitney U test; Fig. 2d). One of the core features of OV is GI. The miGISig score of TCGA-OV patients was calculated, the same used in the TCGA-BC cohort. These patients were then classified into the GU-like and GS-like groups based on the median score of miGISig. There was a similar trend as in BC, with significantly higher CSPM burden and aneuploidy score in the GU-like group compared with that in the GS-like group (median somatic mutations 77 vs. 59.5, P < 0.001; and median aneuploidy score 14.5 vs. 13, P = 0.033; Mann–Whitney U test; Fig. 2e). These results highlighted the association of the miGISig with GI.

### Association between the miGISig and clinical characteristics

We first compared the clinical characteristics between the miGISig-derived high-risk and low-risk groups, and found that ER, PR and TP53 mutations were significantly different (Additional file 1: Table S6). Patients with high miGISig were more likely to be characterized as ER-/PR-negative and high TP53 mutation, whereas patients with ER-/PR-positive and low TP53 mutation rates were enriched in the miGISig-derived low-risk group, suggesting that the miGISig was additionally associated with known prognostic factors. Therefore, to further examine whether the prognostic value of the miGISig was independent of these common clinicopathological factors, the performance of miGISig was tested compared with common clinical variables, using multivariate analysis. The results demonstrated a significant association between miGISig and poor prognosis when adjusted for the various clinical factors in all BC cohorts, indicating that the miGISig was an independent predictor of poor prognosis (Additional file 1: Figure S1).

### Establishment of a composite miRNA-clinical prognostic indicator

**Fig. 3:**
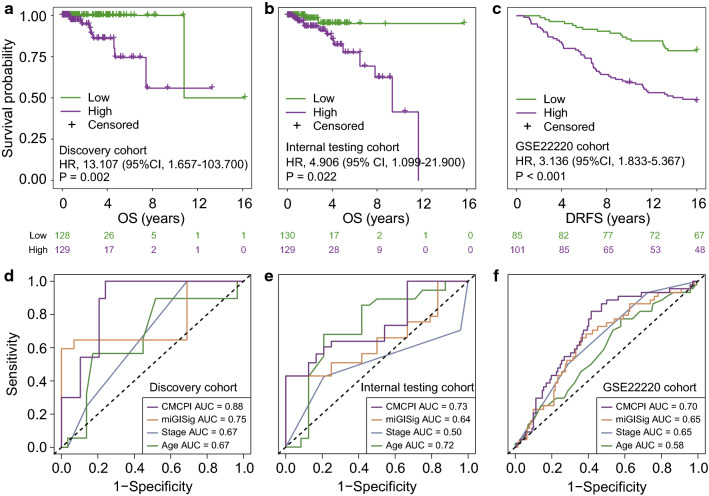
Establishment and performance comparison of CMCPI. Kaplan–Meier estimates of OS or DRFS of patients with low or high CMCPI scores in the discovery cohort (**a**), internal testing cohort (**b**) and GSE22220 cohort (**c**). HRs and 95% CIs for high vs. low miGISig score was estimated using the univariate Cox analysis. P values comparing risk groups were calculated with the log-rank test. The ROC analysis at five years of OS or DRFS for the CMCPI, miGISig, stage and age in the discovery cohort (**d**), internal testing cohort (**e**) and GSE22220 cohort (**f**). *BC* breast cancer, *CIs* confidence intervals, *CMCPI* composite miRNA-clinical prognostic indicator, *DRFS* Distant relapse-free survival, *HRs* Hazard ratios, *OS* overall survival

To further improve the prognostic performance, we combined the miGISig with stage and age to fit a multivariate cox regression model in TCGA discovery cohort and established a composite miRNA-clinical prognostic indicator (CMCPI) calculated as (0.069 × age) + (0.527 × stage) + (1.363 × miGISig score). The median CMCPI score of the discovery cohort was used as a cut-off value for stratifying patients. Remarkably, the CMCPI was significantly associated with prognosis in all BC cohorts, and the CMCPI-derived high-risk group had significantly shorter survival times compared with patients in the CMCPI-derived low-risk group (HR = 13.107, 95% CI 1.657–103.700, P = 0.002 for TCGA discovery cohort; HR = 4.906, 95% CI 1.099–21.900, P = 0.022 for the internal testing cohort, and HR = 3.136, 95% CI 1.833–5.367, P < 0.001 for the GSE22220 cohort; Fig. 3a–c).

We further compared the CMCPI with the miGISig, age and stage in all BC cohorts. The CMCPI achieved significantly improved survival estimation with 5-year ROC AUC of 0.88, 0.73 and 0.70 compared with age, stage and miGISig alone in all BC cohorts, respectively (Fig. 3d–f). These results further strengthened the clinical significance of the miGISig in combination with additional important clinical features, with effective predictive performance in predicting poor prognosis in BC patients.

### Minimally invasive diagnostic value of the miGISig for BC

**Fig. 4:**
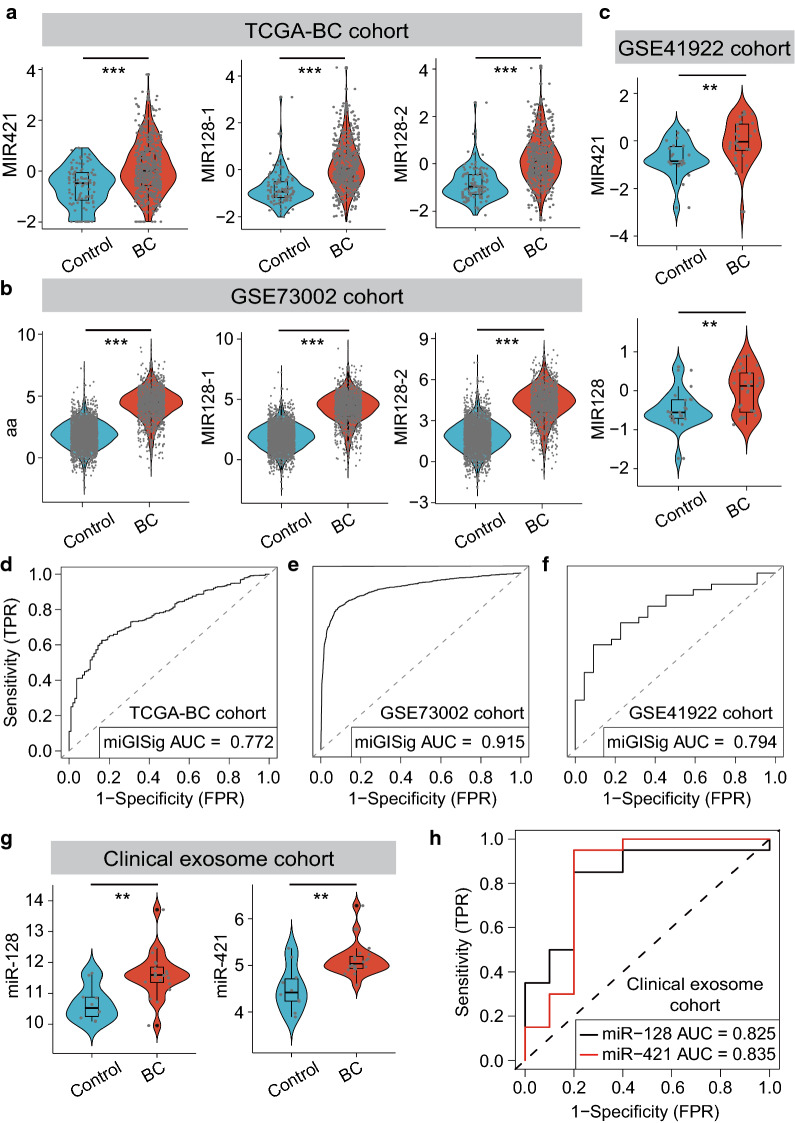
Value of miGISig for the diagnosis of BC. Violin diagram of the miGISig miRNAs expression level in healthy controls and BC patients in TCGA-BC cohort (**a**), GSE73002 cohort (**b**) and GSE41922 cohort (**c**). Statistical analysis was performed using the Mann–Whitney U test. ROC curve for the performance of the miGISig in the diagnosis in the TCGA-BC cohort (**d**), GSE73002 cohort (**e**) and GSE41922 cohort (**f**). **g** Violin diagram of the miGISig miRNAs expression level in healthy controls and BC patients in the in-house clinical exosome cohort. Statistical analysis was performed using the Mann–Whitney U test. **h** ROC curve for the performance of *miR-128* and *miR-421* in the clinical exosome cohort. *AUC* the area under the curve, *BC* breast cancer, *ROC* area under the receiver operating characteristic curve. *P-value < 0.05, **P-value < 0.01, ***P-value < 0.001

In addition, we next examined the performance of the miGISig for risk assessment of BC. We first performed comparative expression analysis for *MIR421*, *MIR128-1* and *MIR128-2* between tumors and healthy controls. The three miRNAs had significantly higher expression in breast tumors compared with that in healthy controls in TCGA, GSE73002 and GSE41922 cohorts (Fig. 4a–c), which indicated their oncogenic roles in BC development and highlighted the potential of the miGISig for the risk assessment of BC. Therefore, we assessed the diagnostic accuracy of the miGISig in detecting BC patients. The miGISig could effectively differentiate BC from healthy controls in TCGA (AUC = 0.772), GSE73002 (AUC = 0.915) and GSE41922 (AUC = 0.794) cohorts, despite the absence of *MIR-128-2* in the GSE41922 cohort (Fig. 4d–f), demonstrating the reliable and robust performance of the miGISig in BC risk prediction.

To further investigate the clinical adaptability of the miGISig as a potential minimally invasive diagnostic biomarker, we next analyzed the expression level of these miRNAs in circulating exosomes from our in-house clinical cohort of 30 samples using small RNA-seq. Compared with that in healthy individuals, the expression level of exosomal *miR-128* and *miR-421* were significantly higher in the serum of BC patients (P = 0.003 for *miR-128* and P = 0.002 for *miR-421*, Mann–Whitney U test; Fig. 4g). In addition, we evaluated the diagnostic value of exosomal *miR-128* and *miR-421* using ROC analysis, which revealed that both exosomal *miR-128* (AUC = 0.825) and *miR-421* (AUC = 0.835) were significant predictors of BC risk (Fig. 4h). Furthermore, the expression levels were also significantly higher in BC patients with stage I for all the miRNAs tested (Figure S2A). BC patients with stage I were distinguished from healthy individuals with an AUC of 0.809 and 0.773 for *miR-421* and *miR-128*, respectively (Additional file 1: Figure S2B). Taken together, our results validated and confirmed the in silico findings from public BC datasets and highlighted that the miGISig could serve as a minimally invasive diagnostic biomarker for early risk assessment of BC in the clinic.

### Overexpression of the miGISig increases genomic instability by inducing an S-phase arrest and promotes the growth of breast cancer cells.

**Fig. 5:**
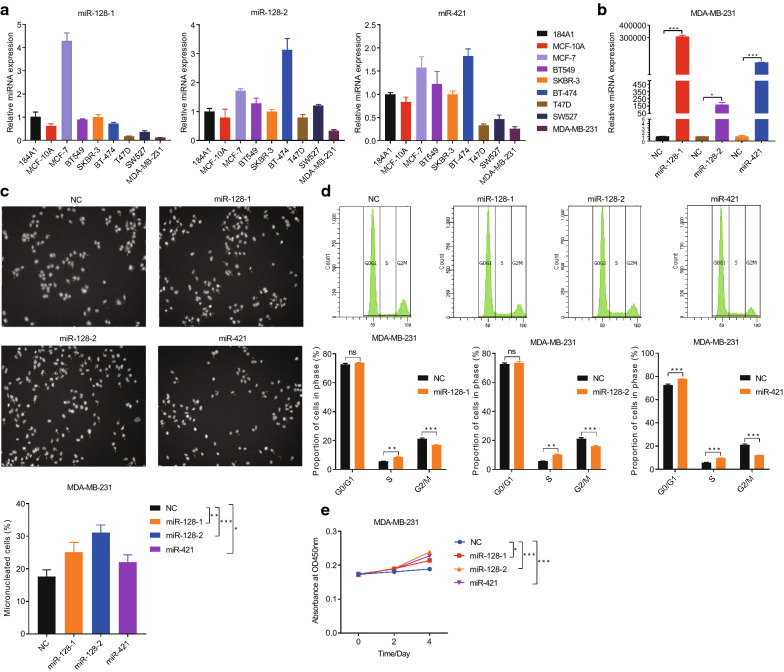
Overexpression of *miR128-1*, *miR128-2* and *miR421* promotes genomic instability, induces an S-phase arrest and promotes the growth of breast cancer cells. QRT-PCR detection of *miR128-1*, *miR128-2* and *miR421* expression in seven breast cancer cells and normal human mammary epithelial cells 184A1 and MCF-10A (**a**). QRT-PCR detection of *miR128-1*, *miR128-2* and *miR421* expression transfected with miRNAs mimics in MDA-MB-231 cells (**b**). The percentage of multinuclei and micronuclei in MDA-MB-231 cells after overexpression of *miR128-1*, *miR128-2* and *miR421* (**c**). Cell cycle distribution of MDA-MB-231 cells after overexpression of *miR128-1*, *miR128-2* and *miR421* (**d**). Cell growth curve of MDA-MB-231 cells with *miR128-1*, *miR128-2* and *miR421* overexpression (**e**)

Since multi-nuclei and micro-nuclei are biomarkers of genotoxic and chromosomal instability [26, 27], we detected the frequency of multi-nuclei and micro-nuclei following overexpression of the three miRNAs. We first detected the expression level of *miR-128*-*1*, *miR-128*-*2*, and *miR-421* in BC cells and healthy human mammary epithelial cells, 184A1 and MCF-10A, and then selected the genomically unstable aneuploid, MDA-MB-231 cell line with a low-level expression of the three miRNAs to perform the following experiments (Fig. 5a). miRNAs mimics were transfected into MDA-MB-231 cells and cultured for 48 h, and the expression level of *miR-128*-*1*, *miR-128*-*2* and *miR-421* was markedly increased (Fig. 5b). Using a high-content system, we showed that overexpression of *miR-128*-*1*, *miR-128*-*2* and *miR-421* spontaneously increased the proportion of multi-nuclei and micro-nuclei in the MDA-MB-231 cell line (25, 31 and 22% in the *miR-128*-*1*, *miR-128*-*2* and *miR-421*, groups, respectively, vs. 18% in the control group) (Fig. 5c). Since GI can result from defects in cell cycle regulation [28–33], we examined whether the overexpression *miR-128*-*1*, *miR-128*-*2* and *miR-421* lead to the aberrant cell cycle progression. FACS analysis showed that the proportion of cells in the S phase increased, while the number of cells in G_2_/M phase decreased following the overexpression of the three miRNAs using mimics in the MDA-MB-231 and MCF-7 cell lines (Fig. 5d and Additional file 1: Figure S3A and S3B). Consistent with these results, the CCK-8 assay demonstrated that the proliferative ability of the MDA-MB-231 and MCF-7 cell lines was significantly increased following the overexpression of *miR-128*-*1*, *miR-128*-*2* and *miR-421* (Fig. 5e and Additional file 1: Figure S3C). These results demonstrated that the miGISig was associated with GI and could serve as oncogenes to promote the growth of breast cancer cells.

**Fig. 6:**
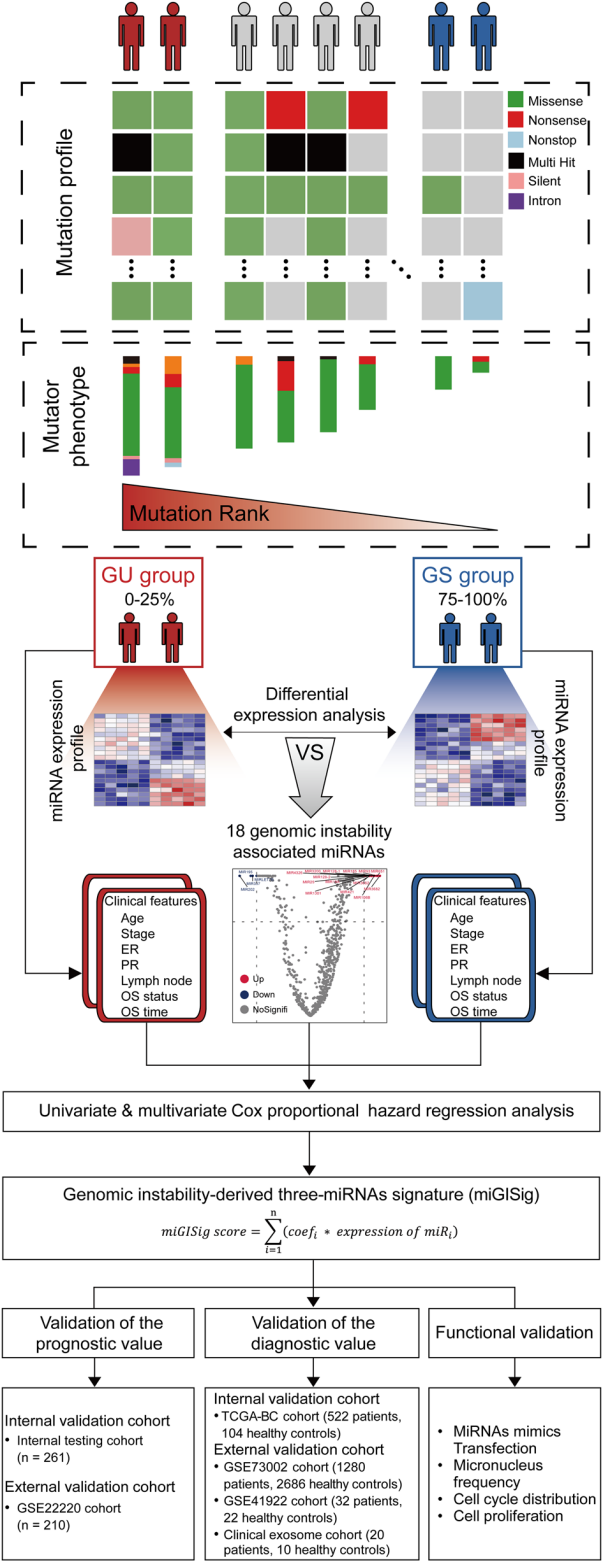
Study flowchart. The study was performed in multicenter cohorts, including TCGA-BC, GSE22220, GSE73002, GSE41922, and in-house clinical exosome cohorts. Genome instability-derived miRNAs signature (miGISig) was identified in the discovery cohort. The miGISig was applied to an internal validation cohort and multiple external validation cohorts to verify its value in prognosis and diagnosis of BC. The effects of the miGISig on BC growth were investigated using the in vitro functional assays. *BC* breast cancer, *GI* Genome instability

## Discussion

BC is the most frequently diagnosed cancer and the leading cause of cancer-associated death in women. Considerable efforts have been made in the development of precision medicine for BC; however, its early diagnosis and prognosis risk stratification, using a minimally invasive method is still a clinical challenge. In this study, we focused on miRNAs involved in GI, as evaluated by expression alteration and tumor mutator phenotype and identified 18 candidate GI-related miRNAs. Target genes of the 18 candidate GI-related miRNAs were enriched in known GI pathways. For example, TGF-β maintains genomic stability by enhancing the DNA damage response [34]. Activation of the PI3K/Akt pathway can support tumor growth and progression, and can lead to inhibition of DNA repair, which may contribute to GI. Recent studies have indicated that sustained mitogen-activated protein kinase signaling relaxes the cell cycle checkpoints and allows cells to escape prolonged G_2_ arrest by inducing the accumulation of the pro-mitotic kinase, thereby enhancing GI [35]. DNA damage response (DDR) assures the maintenance of GI, while a recent report has shown that Notch is a direct negative regulator of DDR.

Furthermore, the 18 candidate GI-related miRNAs can differentiate BC patients into two clusters with significantly different prognosis, *UBQLN4* expression and aneuploidy score. A recent study indicated that *UBQLN4* is an essential driver of GI and its overexpression represses homologous recombination-mediated double-strand break repair in aggressive tumors [36]. The aneuploidy score reflects the total burden of arm-level copy-number alterations and is an indicator of chromosome instability [37, 38]. These results provided evidence to support the relevance of the 18 miRNAs in GI. The identification of these miRNAs involved in GI will not only enhance our understanding of the biology of GI, but it will also provide new candidates for early diagnosis and prognosis of BC.

By focusing on these miRNAs involved in GI, we identified three oncogenic miRNAs (*MIR421*, *MIR128-1* and *MIR128-2*), with the independent prognostic value from the list of 18 candidate GI-related miRNAs and produced a three-miRNA signature, termed miGISig, which is predictive of GI and clinical outcome. The predictive value of miGISig was validated by its association with clinical outcome and GI in TCGA and other public BC datasets across different platforms. Furthermore, the miGISig is not only an independent predictor, but also exerts superior or comparable performance to other clinical factors. We further leveraged the complementary value of molecular and clinical characteristics and showed that combining the miGISig with several complementary clinical factors could provide a more accurate estimation of prognosis in BC. For the miGISig identified here, *MIR421* has been reported to cause the failure of DNA repair by suppressing the expression of ataxia-telangiectasia mutated, a core component of the DNA repair system, thus enhancing GI [39]. Furthermore, the overexpression of *MIR421* has been previously shown to promote cancer cell proliferation in BC and in non-small cell lung cancer [40, 41]. *MIR128-1* and *MIR128-2*, members of the miRNA *MIR128* family, encoded the same mature *miR-128* whose expression pattern and roles in tumorigenesis and development vary in different types of cancer. However, little is known about their association with GI in BC. Our in *vitro* functional assays suggested that overexpression of the miGISig increases GI, induces an S-phase arrest and promotes the growth of breast cancer cells. Many studies have suggested that aberrant miRNA expression is not only involved in cancer prognosis, but is also an early event in tumorigenesis [42–44]. In addition, miRNA levels have high stability and activity, which enables their detection in the serum, plasma, and other body fluids, using RT-qPCR, miRNA microarrays and deep sequencing techniques [20], highlighting the superiority of circulating miRNAs, as minimally invasive biomarkers in cancers. However, the mechanisms and biological impact of circulating miRNAs still remain unknown. Exosomes are cell-driven vesicles, which carry nucleic acids, proteins, lipids, and metabolites from their host cells [45]. Exosomes can be isolated from readily available biological fluids, such as blood and urine, and this minimally invasive advantage presents an attractive novel biomarker for diagnostic applications [46]. For the miRNA markers identified here, little is known with respect to their potential for minimally invasive diagnosis of BC. Therefore, we integrated circulating exosomal miRNA profiles from TCGA and GEO tissue datasets and found that the miRNAs in the miGISig were all upregulated in both BC tissues and circulating exosomes when compared with normal breast tissues or circulating exosomes from healthy subjects. Furthermore, the miGISig revealed higher efficacy and stability in distinguishing BC from healthy subjects. Collectively, these results suggested that the miGISig was a GI-derived oncogenic maker that represents a promising minimally invasive clinical genomic tool for BC detection and prognosis; however, additional cohorts are required to validate these findings. Furthermore, the miGISig identified in the EVs provided further evidence supporting exosome-mediated delivery of oncogenic miRNAs is associated with BC carcinogenesis and prognosis. From a therapeutic perspective, identification of oncogenic GI-associated miRNAs in EVs also implied potential therapeutic strategy via transfecting anti-miRNA compounds or miRNA-based agents into exosomes to interfere with the load or delivery of these oncogenic exo-miRNAs for targeting genomic instability as a multimodality treatment strategy of BC.

## Conclusions

In summary, using genome-wide miRNA expression profiles from the tumor, healthy tissues and circulating exosomes, our study indicated the clinical value of GI-associated miRNAs and established a GI-derived three-miRNA signature allowing early detection and prognostic risk stratification with minimal invasion for BC. Following further investigation in prospective cohort studies, our study highlighted the potential clinical value of the EVs-derived GI-associated miRNAs, as a minimally invasive genomic tool to improve BC precision medicine.

## Methods

### Study design

The overall research design was illustrated in Fig. 6. The study was performed using multicenter prospective cohorts, including The Cancer Genome Atlas (TCGA)-BC, GSE22220, GSE73002, GSE41922, and in-house clinical exosome cohorts. miGISig was identified in the discovery cohort. The miGISig was then applied to an internal validation cohort and multiple external validation cohorts to verify its value in the prognosis and diagnosis of BC. The effects of the miGISig on BC growth were investigated using in vitro functional assays.

### Public RNA-sequencing (Seq), microarray and clinical data collection

Clinical characteristics, miRNA-seq [Illumina HiSeq reads per million (RPM) type] expression data, RNA-seq (Illumina Hiseq fragments per kilobase of transcript per million type) expression data and somatic mutation information from patients with breast tumors and ovarian cancers (OV) were obtained from TCGA Genomic Data Commons Data Portal (https://portal.gdc.cancer.gov/). Only data from female patients, including paired miRNA, mRNA expression profiles, survival information and somatic mutation information, were retained. A total of 522 BC samples and 355 OV samples, using the aforementioned information, were retained for further investigation. The non-TCGA BC miRNAs microarray datasets used in the validation phase were downloaded from the Gene Expression Omnibus (GEO) database (https://www.ncbi.nlm.nih.gov/geo/), including GSE22220 (n = 210) [47], GSE73002 (n = 3966) [48] and GSE41922 (n = 54) [49]. Public BC miRNA datasets used in this study were listed in Additional file 1: Tables S1 and S2.

### Plasma exosomal dataset

A total of 30 subjects, including 20 BC patients and 10 age-matched healthy women were enrolled from the Cancer Hospital, Chinese Academy of Medical Sciences (CHCAMS) for this study. Peripheral blood samples (10 ml) from these 30 subjects were collected for exosome isolation, which was confirmed by nanoparticle tracking analysis (NTA), transmission electron microscopy (TEM), and western blot analysis according to previously reported protocols [50]. Small RNA-seq was performed using the Illumina HiSeq 2000 platform according to the manufacturer’s protocol and miRNA expression levels were calculated using the reads per million (RPM) values. This study was approved by the Ethics Committee of the CHCAMS and written informed consent was provided by all the participants.

### Establishment of a miGISig

The GI for each TCGA sample was measured using the cumulative somatic point mutation (CSPM) burden within a cancer genome. Tumors with a high CSPM burden (ranked within the top 25%) were defined as the genomically unstable (GU)-like group and those with a low CSPM burden (within the last 25%) were defined as the genomically stable (GS)-like group. The differentially expressed miRNAs between GU-like and GS-like groups were defined as candidate GI-associated miRNAs (GImiRs). Prognostic GImiRs were identified using the univariate Cox regression analysis for overall survival (OS) time. Finally, the miGISig was constructed as follows:

$$miGISig = \sum\limits_{i = 1}^{N} {expression\,of\,miRNA_{i} * Coefficient_{i} }$$

where $N$ is the number of prognostic GImiRs, expression of miRNA_i_ is the expression value of prognostic GImiR_i_, and Coefficient_i_ is the estimated regression coefficient of GImiR_i_ in the multivariable Cox regression analysis.

The optimal cut-off value for risk stratification was determined using the point representing the 100% true-positive rate and 0% false-positive rate in the receiver operating characteristics (ROC) curve, in the discovery cohort.

### Cell culture and transfection

The MCF-7, BT549, SKBR-3, BT474, T47D, SW527 and MDA-MB-231 breast cancer cell lines, and the 184A1 and MCF-10A normal human mammary epithelial cell lines were purchased from the American Type Culture Collection (ATCC; Rockville, MD, USA). The 184A1 and MCF-10A cell lines were maintained in DMEM-F12 containing 2 mM l-glutamine, 20 ng/ml EGF, 100 ng/ml cholera toxin, 0.01 mg/ml insulin, 500 ng/ml hydrocortisone and 5% horse serum (HyClone, USA). The MCF-7 cells were maintained in DMEM containing 2 mM l-glutamine, 1 mM sodium pyruvate, 10 mM HEPES and 10% FBS (Gibco, USA). The BT549 and SKBR-3 cells were cultured in RPMI-1640 supplemented with 10% FBS, while the BT474 and T47D cells were maintained in RPMI-1640 containing 2 mM l-glutamine, 4.5 g/l glucose, 1.5 g/l sodium bicarbonate, 1 mM sodium pyruvate, 0.01 mg/ml insulin, 10 mM HEPES and 5% FBS. The SW527 cells were maintained in DMEM containing 2 mM l-glutamine, 4.5 g/l glucose, 1.5 g/l sodium bicarbonate, and 10% FBS. All the cell lines were maintained at 37 °C in a humidified atmosphere with 5% CO_2_, while the MDA-MB-231 cells were maintained in Leibovitz’s L-15 medium with 10% FBS at 37 °C.

### miRNA mimics transfection

Sequences of *miR-128*-*1-5p*, *miR-128*-*2-5p* and *miR-421* were obtained from the miRBase database. The miRNAs mimics and negative controls were designed and synthesized from RiboBio (Guangzhou, China). The primer sequences of the miRNAs and controls were listed in Additional file 1: Table S3. Transfections for miRNAs mimics were performed using HiperFect (Qiagen, Valencia, CA, USA) according to the manufacturers' instructions.

### Reverse transcription-quantitative PCR (RT-qPCR)

Total RNA was isolated using the TRIzol® reagent (Thermo Scientific, Grand Island, NY, USA) and then reverse transcribed into cDNA using a Quantscript RT kit (Tiangen, Beijing, China). The RT-qPCR analysis was performed using a StepOnePlus Real-Time PCR system (Applied Biosystems, Foster City, CA, USA) according to standard procedures. The relative expression levels of the miRNA were normalized to that of U6, which served as an endogenous control.

### Cell proliferation assays

The MCF-7 and MDA-MB-231 cell lines were plated into 96-well microplates at 1 × 10^3^ cells per well. At the indicated time points, the viability of the cells was determined using a Cell Counting Kit-8 (CCK8, Dojindo) and measured at 450 nm, with the BioTek Gen5 system (BioTeck, USA). The experiments were repeated three times, with the representative experiment shown in the figures.

### Flow cytometry (FACS) analysis

The MCF-7 and MDA-MB-231 cell lines were plated into 6-well microplates, and cells at 40–50% confluency were transfected with miRNAs mimics and cultured for 48 h (h). The collected cells were incubated with the cell cycle kit (Beckman Coulter, Pasadena, CA, USA) according to the manufacturer's instructions and the results were analyzed using flow cytometry (Beckman Coulter, Pasadena, CA, USA).

### Immunofluorescence staining

Cells were seeded into 6-well microplates, and cells at 40–50% confluency were transfected with miRNAs mimics and cultured for 24 h. Then, the cells were re-seeded in a plate and cultured for 24 h. Cells were fixed with 4% paraformaldehyde and permeabilized with Triton X-100 (Sigma-Aldrich, St. Louis, MO, USA), and stained with DAPI (Shanghai Yeasen Biotechnology Co. Ltd. Shanghai, China) for 5 min. Then, the cells were imaged using high content analysis (HCA, Thermo Scientific, USA).

### Construction of an integrated prognostic index

Multivariate cox regression analysis was applied to construct an integrated prognostic index by combining age, stage and miGISig. The median score was used for the cut-off value for the integrated prognostic index. Time-dependent ROC curve was used to compare the prognostic performance among age, stage, miGISig and the integrated prognostic index.

### Statistical analysis

All statistical analyses were performed using the R software (V.3.4.4) with the following packages: ‘samr’, ‘ggpubr’, ‘survival’, ‘survminer’, ‘timeROC’ and ‘ROCit’. The Mann–Whitney U test was used to compare continuous variables. Survival analysis was conducted using the Kaplan–Meier method and the log-rank test. Univariate and multivariate analyses with Cox proportional hazards regression for OS time were performed on the individual variables by calculating hazard ratios (HR) and 95% confidence intervals (CI). Differential expression analysis was performed using the Significance Analysis of Microarrays method, and fold change > 1.5 or < 0.67 and false discovery rate adjusted P < 0.05 was considered significant. The time-dependent ROC curve was calculated with the nearest neighbor estimation method. The unsupervised hierarchical clustering analysis was performed with Euclidean distance for determining the similarity between patients and the ward's linkage method for merging similar objects.

### Target genes and functional enrichment analysis of miRNAs

Experimentally verified miRNA-target relationships were obtained from The Encyclopedia of RNA Interactomes (ENCORI, http://starbase.sysu.edu.cn/) [51]. Pearson correlation coefficients were computed to measure the correlation between miRNAs and mRNAs. Experimentally verified miRNA targets with correlation coefficient < − 0.3 and P-value < 0.05 were selected as BC-specific miRNA targets. Functional enrichment analysis was conducted for BC-specific miRNA targets to determine significantly enriched biological processes (BP) terms in Gene Ontology (GO) and Kyoto Encyclopedia of Genes and Genomes (KEGG) pathway using DAVID Bioinformatics Resources 6.8 (version 6.8; https://david.ncifcrf.gov/) [52].

## Supplementary Information

**Additional file 1: Table S1.** Public miRNAs datasets used in this study. **Table S2.** Clinicopathological characteristics of BC patients used in this study. **Table S3.** Primer sequences of three genomic instability-related oncogenic miRNAs and controls in this study. **Table S4.** Lists of 18 genomic instability-related miRNAs in BC. **Table S5.** Univariate Cox regression analyses of three genomic instability-related miRNAs associated with overall survival in BC. **Table S6.** Comparision of clinical characterics between miGISig-derived high-risk and low-risk groups. **Figure S1.** Multivariate analysis of the miGISig with clinical characteristics in different cohorts. **Figure S2.** Value of miGISig for early diagnosis of BC. **Figure S3.** Overexpression of *miR128-1*, *miR128-2* and *miR421* induces a S-phase arrest and promotes the proliferative ability of MCF-7 cells.

## Data Availability

The dataset analyzed during the current study are available in the TCGA (https://portal.gdc.cancer.gov/) and GEO databases (https://www.ncbi.nlm.nih.gov/geo/query/acc.cgi?acc=GSE22220, https://www.ncbi.nlm.nih.gov/geo/query/acc.cgi?acc=GSE73002, https://www.ncbi.nlm.nih.gov/geo/query/acc.cgi?acc=GSE41922).

## Abbreviations

BC: Breast cancer
CI: Confidence intervals
CSPM: Cumulative somatic point mutation
ENCORI: Encyclopedia of RNA interactomes
ER: Estrogen receptor
EVs: Extracellular vesicles
GEO: Gene Expression Omnibus
GI: Genomic instability
GO: Gene Ontology
GU: Genomically unstable
GS: Genomically stable
miRNA: MicroRNA
HER2: Human epidermal growth factor receptor 2
PR: Progesterone receptor
TCGA: The Cancer Genome Atlas
KEGG: Kyoto Encyclopedia of Genes and Genomes
OS: Overall survival
OV: Ovarian cancers
RPM: Reads per million
ROC: Receiver operating characteristics
