# Supplementary material for: Genomic instability-derived plasma extracellular vesicle-microRNA signature as a minimally invasive predictor of risk and unfavorable prognosis in breast cancer
Source: J Nanobiotechnology. 2021 Jan 12;19:22. doi: 10.1186/s12951-020-00767-3 (PMC7802300; doi:10.1186/s12951-020-00767-3)
Supplement: Supplementary file 1 — Additional file 1: Table S1. Public miRNAs datasets used in this study. Table S2. Clinicopathological characteristics of BC patients used in this study. Table S3. Primer sequences of three genomic instability-related oncogenic miRNAs and controls in this study. Table S4. Lists of 18 genomic instability-related miRNAs in BC. Table S5. Univariate Cox regression analyses of three genomic instability-related miRNAs associated with overall survival in BC. Table S6. Comparision of clinical characterics between miGISig-derived high-risk and low-risk groups. Figure S1. Multivariate analysis of the miGISig with clinical characteristics in different cohorts. Figure S2. Value of miGISig for early diagnosis of BC. Figure S3. Overexpression of miR128-1, miR128-2 and miR421 induces a S-phase arrest and promotes the proliferative ability of MCF-7 cells. [file 12951_2020_767_MOESM1_ESM.docx]

**Additional Information**

**Table S1. Public miRNAs datasets used in this study**

| Datasets | Use | Platform | No. of patients | No. of healthy controls | Survival |
| --- | --- | --- | --- | --- | --- |
| TCGA-BC | Discovery & Internal test | Illumina HiSeq | 522 | 104 | OS |
| TCGA-OV | External validation | Illumina HiSeq | 355 | - | OS |
| GSE22220 | External validation | Illumina Human v1 MicroRNA expression beadchip | 210 | - | DRFS |
| GSE73002 | External validation | 3D-Gene Human miRNA V20_1.0.0 | 1280 | 2686 | - |
| GSE41922 | External validation | Exiqon LNA RT-PCR Human panels (1 & 2) | 32 | 22 | - |

BC, breast cancer; DRFS, Distant relapse-free survival; OS, Overall survival; OV, ovarian cancer

**Table S2. Clinicopathological characteristics of BC patients used in this study**

| Covariates |  | TCGA-BC cohort | Discovery cohort | Internal testing cohort | P value | GSE22220 cohort | GSE41922 cohort |
| --- | --- | --- | --- | --- | --- | --- | --- |
|  |  | (N = 522) | (N = 261) | (N = 261) |  | (N = 210) | (N = 32) |
| Age, years (mean ± SD) |  | 58.6 ± 12.6 | 58.8 ± 13.1 | 58.4 ± 12.2 | ${\text{0.769}\text{ }}^{a}$ | 54.4 ± 10.7 | 50.2 ± 13.2 |
| lymph nodes, counts (mean ± SD) |  | 10.3 ± 9.1 | 11.0 ± 9.6 | 9.7 ± 8.5 | ${0.134}^{a}$ | 1.7 ± 3.2 | n.a. |
| ER, no (%) | Positive | 386(74.0) | 199(76.2) | 187(71.7) | ${0.316}^{b}$ | 128(61.0) | 23(71.9) |
|  | Negative | 116(22.2) | 53(20.3) | 63(24.1) |  | 82(39.0) | 9(28.1) |
|  | Unknown | 20(3.8) | 9(3.5) | 11(4.2) |  | 0(0.0) | 0(0.0) |
| PR, no (%) | Positive | 339(65.0) | 171(65.5) | 168(64.4) | $1^{b}$ | n.a. | n.a. |
|  | Negative | 162(31.0) | 81(31.0) | 81(31.0) |  | n.a. | n.a. |
|  | Unknown | 21(4.0) | 9(3.5) | 12(4.6) |  | n.a. | n.a. |
| HER2, no (%) | Positive | 55(10.5) | 23(8.8) | 32(12.3) | ${0.268}^{b}$ | n.a. | 14(43.7) |
|  | Negative | 276(52.9) | 141(54.0) | 135(51.7) |  | n.a. | 11(34.4) |
|  | Unknown | 191(36.6) | 97(37.2) | 94(36.0) |  | n.a. | 7(21.9) |
| TP53 mutation, no (%) | mut | 156(29.9) | 75(28.7) | 81(31.0) | ${0.633}^{b}$ | n.a. | n.a. |
|  | wt | 366(70.1) | 186(71.3) | 180(69.0) |  | n.a. | n.a. |
| Stage, no (%) | I | 98(18.8) | 55(21.1) | 43(16.5) | ${0.375}^{b}$ | n.a. | n.a. |
|  | II | 298(57.1) | 139(53.3) | 159(60.9) |  | n.a. | n.a. |
|  | III | 118(22.6) | 62(23.8) | 56(21.4) |  | n.a. | n.a. |
|  | IV | 2(0.3) | 1(0.3) | 1(0.4) |  | n.a. | n.a. |
|  | Unknown | 6(1.2) | 4(1.5) | 2(0.8) |  | n.a. | n.a. |
| Vital status, no (%) | Alive | 495(94.8) | 250(95.8) | 245(93.9) | ${0.324}^{b}$ | n.a. | n.a. |
|  | Dead | 27(5.2) | 11(4.2) | 16(6.1) |  | n.a. | n.a. |
| Relapse, no (%) | with | n.a. | n.a. | n.a. |  | 131(62.4) | n.a. |
|  | without | n.a. | n.a. | n.a. |  | 79(37.6) | n.a. |
| Grade, no (%) | I | n.a. | n.a. | n.a. |  | 42(20.0) | n.a. |
|  | II | n.a. | n.a. | n.a. |  | 81(38.6) | n.a. |
|  | III | n.a. | n.a. | n.a. |  | 63(30.0) | n.a. |
|  | Unknown | n.a. | n.a. | n.a. |  | 24(11.4) | n.a. |

^a^ Mann-Whitney U test

^b^ Chi square test

n.a., not available

**Table S3. Primer sequences of three genomic instability-related oncogenic miRNAs and controls in this study**

| Primer Name | Forward Primer Sequence (5' to 3') | Reverse Primer Sequence (5' to 3') | Used for |
| --- | --- | --- | --- |
| miR-128-1-5p | ACACTCCAGCTGGGCGGGGCCGTAGCACTGT | CTCAACTGGTGTCGTGGAGTCGGCAATTCAGTTGAGTCTCAGAC | qRT-PCR |
| miR-128-2-5p | ACACTCCAGCTGGGGGGGGCCGATACACTCT | CTCAACTGGTGTCGTGGAGTCGGCAATTCAGTTGAGTCTCGTAC | qRT-PCR |
| miR-421 | ACACTCCAGCTGGGATCAACAGACATTAATT | CTCAACTGGTGTCGTGGAGTCGGCAATTCAGTTGAGGCGCCCAA | qRT-PCR |
| U6 | CTCGCTTCGGCAGCACA | AACGCTTCACGAATTTGCGT | qRT-PCR |

**Table S4. Lists of 18 genomic instability-related miRNAs in BC**

| Stem-loop sequence | Accession | Previous IDs | Symbol |
| --- | --- | --- | --- |
| hsa-let-7b | MI0000063 | hsa-let-7bL | HGNC:MIRLET7B |
| hsa-mir-106b | MI0000734 | - | HGNC:MIR106B |
| hsa-mir-128-1 | MI0000447 | hsa-mir-128a | HGNC: MIR128-1 |
| hsa-mir-128-2 | MI0000727 | hsa-mir-128b | HGNC: MIR128-2 |
| hsa-mir-1301 | MI0003815 | - | HGNC:MIR1301 |
| hsa-mir-185 | MI0000482 | - | HGNC:MIR185 |
| hsa-mir-195 | MI0000489 | - | HGNC:MIR195 |
| hsa-mir-19a | MI0000073 | - | HGNC:MIR19A |
| hsa-mir-202 | MI0003130 | - | HGNC:MIR202 |
| hsa-mir-25 | MI0000082 | - | HGNC:MIR25 |
| hsa-mir-3200 | MI0014249 | - | HGNC:MIR3200 |
| hsa-mir-337 | MI0000806 | - | HGNC:MIR337 |
| hsa-mir-3662 | MI0016063 | - | HGNC:MIR3662 |
| hsa-mir-3682 | MI0016083 | - | HGNC:MIR3682 |
| hsa-mir-421 | MI0003685 | - | HGNC: MIR421 |
| hsa-mir-4326 | MI0015866 | - | HGNC:MIR4326 |
| hsa-mir-651 | MI0003666 | - | HGNC:MIR651 |
| hsa-mir-93 | MI0000095 | hsa-mir-93-7.1;hsa-mir-93-1 | HGNC:MIR93 |

**Table S5. Univariate Cox regression analyses of three genomic instability-related miRNAs associated with overall survival in BC**

| Stem-loop sequence | Accession | Previous IDs | Symbol | Coefficient | Hazard ratio | 95% CI | P-value |
| --- | --- | --- | --- | --- | --- | --- | --- |
| hsa-mir-128-1 | MI0000447 | hsa-mir-128a | HGNC: MIR128-1 | 0.639 | 1.895 | 1.050-3.418 | 0.034 |
| hsa-mir-128-2 | MI0000727 | hsa-mir-128b | HGNC: MIR128-2 | 0.636 | 1.889 | 1.026-3.476 | 0.041 |
| hsa-mir-421 | MI0003685 | - | HGNC: MIR421 | 0.66 | 1.935 | 1.092-3.429 | 0.024 |

**Table S6. Comparision of clinical characterics between** **miGISig-derived high-risk and low-risk groups**

|  |  | Discovery cohort | | | Internal testing cohort | | | GSE22220 cohort | | |
| --- | --- | --- | --- | --- | --- | --- | --- | --- | --- | --- |
| Covariates | | High | Low | P-value | High | Low | P-value | High | Low | P-value |
|  |  | (N = 46) | (N = 215) |  | (N = 47) | (N = 214) |  | (N = 58) | (N = 152) |  |
| Age, years (mean ± SD) | | 55.3 ± 12.4 | 59.5 ± 13.1 | ${0.037}^{a}$ | 58.4 ± 11.5 | 58.4 ± 12.3 | ${0.946}^{a}$ | 55.7 ± 10.8 | 53.9 ± 10.7 | ${0.293}^{a}$ |
| lymph nodes, counts (mean ± SD) | | 12.1 ± 10.1 | 10.7 ± 9.5 | $\text{0.294 }^{a}$ | 8.2 ± 7.2 | 10.1 ± 8.8 | ${0.304}^{a}$ | 2.2 ± 4.0 | 1.5 ± 2.9 | ${0.878}^{a}$ |
| ER, no (%) | Positive | 23(50.0) | 176(81.9) | $\text{1.19E-06 }^{b}$ | 19(40.4) | 168(78.5) | ${\text{5.}\text{31}\text{E-09 }}^{b}$ | 37(63.8) | 91(59.9) | ${0.717}^{b}$ |
|  | Negative | 22(47.8) | 31(14.4) |  | 28(59.6) | 35(16.4) |  | 21(36.2) | 61(40.1) |  |
|  | Unknown | 1(2.2) | 8(3.7) |  | 0(0) | 11(5.1) |  | 0(0) | 0(0) |  |
| PR, no (%) | Positive | 15(32.6) | 156(72.6) | $\text{1.19E-07 }^{b}$ | 17(36.2) | 151(70.6) | ${\text{9.00}\text{E-0}\text{7}\text{ }}^{b}$ | n.a. | n.a. |  |
|  | Negative | 30(65.2) | 51(23.7) |  | 30(63.8) | 51(23.8) |  | n.a. | n.a. |  |
|  | Unknown | 1(2.2) | 8(3.7) |  | 0(0) | 12(5.6) |  | n.a. | n.a. |  |
| HER2, no (%) | Positive | 5(10.9) | 18(8.4) | $\text{0.732 }^{b}$ | 4(8.5) | 28(13.1) | ${\text{0.794}\text{ }}^{b}$ | n.a. | n.a. |  |
|  | Negative | 23(50.0) | 118(54.9) |  | 22(46.8) | 113(52.8) |  | n.a. | n.a. |  |
|  | Unknown | 18(39.1) | 79(36.7) |  | 21(44.7) | 73(34.1 |  | n.a. | n.a. |  |
| TP53 mutation, no (%) | mut | 30(65.2) | 45(20.9) | $\text{5.07E-09 }^{b}$ | 26(55.3) | 55(25.7) | ${\text{1.45}\text{E-04 }}^{b}$ | n.a. | n.a. |  |
|  | wt | 16(34.8) | 170(79.1) |  | 21(44.7) | 159(74.3) |  | n.a. | n.a. |  |
| Stage, no (%) | I | 6(13.0) | 49(22.8) | $\text{0.471 }^{b}$ | 8(17.0) | 35(16.4) | ${\text{0.208}\text{ }}^{b}$ | n.a. | n.a. |  |
|  | II | 26(56.5) | 113(52.5) |  | 28(59.6) | 131(61.2) |  | n.a. | n.a. |  |
|  | III | 13(28.3) | 49(22.8) |  | 10(21.3) | 46(21.5) |  | n.a. | n.a. |  |
|  | IV | 0(0) | 1(0.5) |  | 1(2.1) | 0(0) |  | n.a. | n.a. |  |
|  | Unknown | 1(2.2) | 3(1.4) |  | 0(0) | 2(0.9) |  | n.a. | n.a. |  |
| Vital status, no (%) | Alive | 40(87.0) | 210(97.7) | $\text{0.004 }^{b}$ | 40(85.1) | 205(95.8) | ${\text{0.015}\text{ }}^{b}$ | n.a. | n.a. |  |
|  | Dead | 6(13.0) | 5(2.3) |  | 7(14.9) | 9(4.2) |  | n.a. | n.a. |  |
| Relapse, no (%) | with | n.a. | n.a. |  | n.a. | n.a. |  | 27(46.6) | 52(34.2) | ${0.136}^{b}$ |
|  | without | n.a. | n.a. |  | n.a. | n.a. |  | 31(53.4) | 100(65.8) |  |
| Grade, no (%) | I | n.a. | n.a. |  | n.a. | n.a. |  | 12(20.7) | 30(19.7) | ${0.850}^{b}$ |
|  | II | n.a. | n.a. |  | n.a. | n.a. |  | 24(41.4) | 57(37.5) |  |
|  | III | n.a. | n.a. |  | n.a. | n.a. |  | 16(27.6) | 47(30.9) |  |
|  | Unknown | n.a. | n.a. |  | n.a. | n.a. |  | 6(10.3) | 18(11.9) |  |

^a^ Mann–Whitney U test

^b^ Chi square test

n.a., not available

**
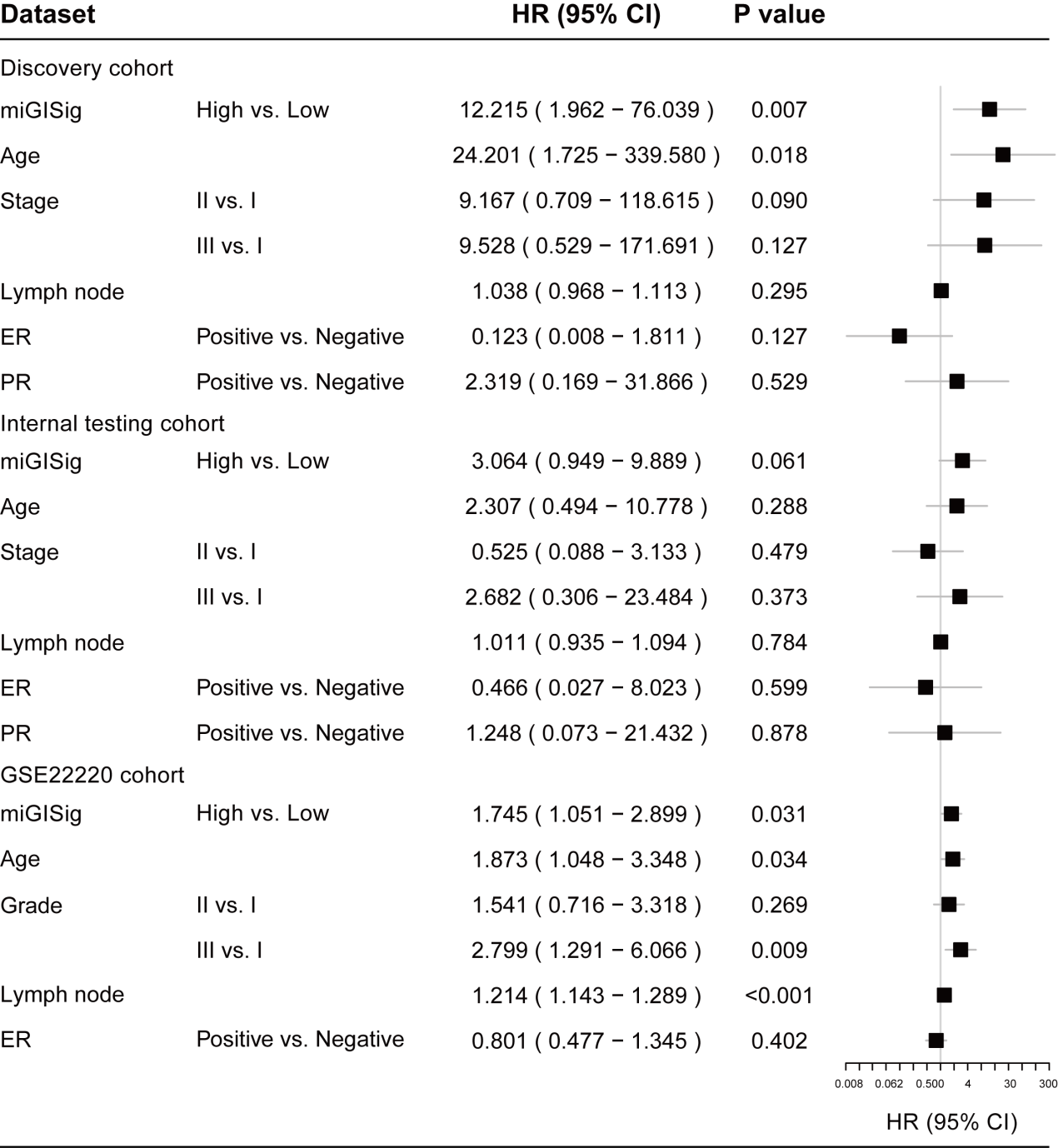
**

**Figure S1.** **Multivariate analysis of the miGISig with clinical characteristics in different cohorts.**

**
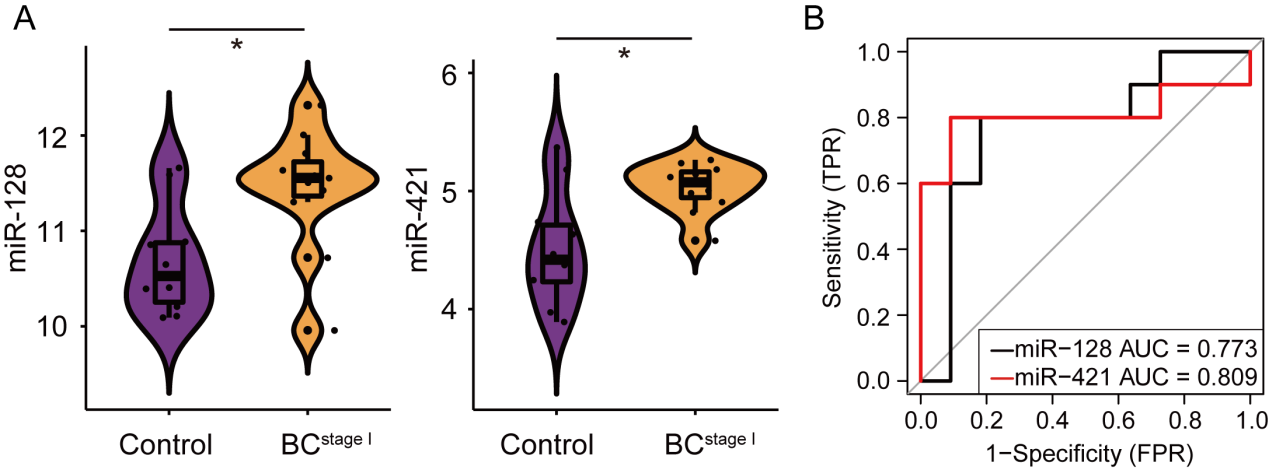
**

**Figure S2. Value of miGISig for early diagnosis of BC.** (A) Violin diagram of the miGISig miRNAs expression level in healthy controls and stage I BC patients in clinical exosome cohort. (B) ROC curve for the performance of miGISig miRNAs in the clinical exosome cohort. AUC, the area under the curve; BC, breast cancer; ROC, area under the curve. * P value < 0.05.


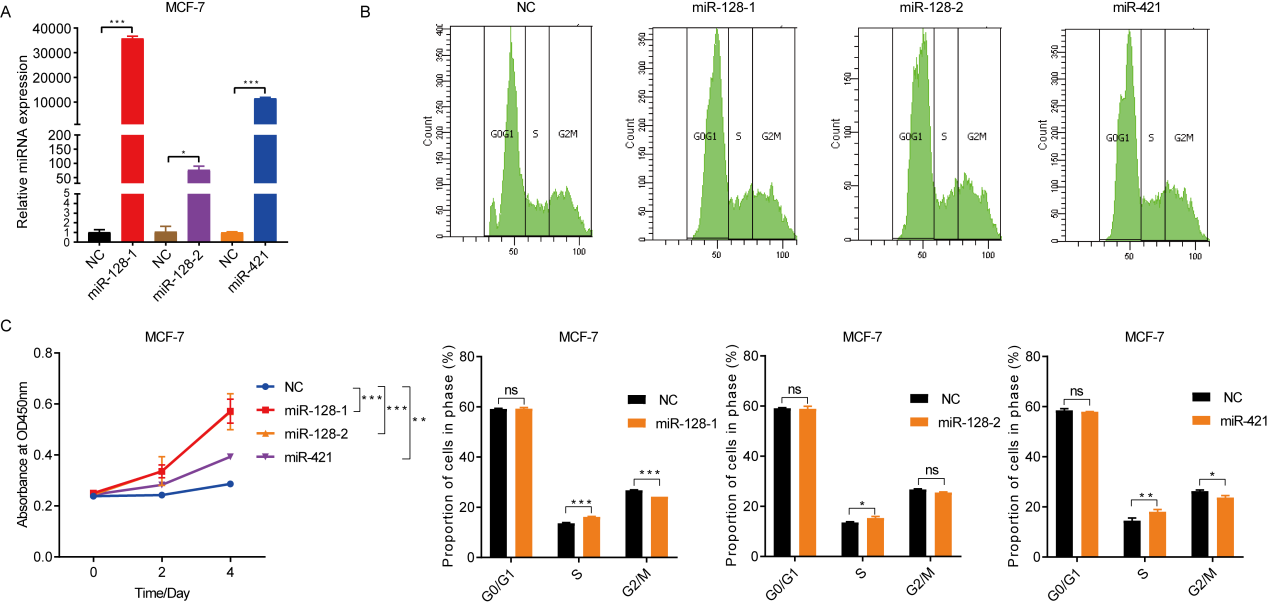


**Figure S3. Overexpression of *miR128-1*, *miR128-2* and *miR421* induces a S-phase arrest and promotes the proliferative ability of MCF-7 cells.** **(A)** qRT-PCR detection of *miR128-1*, *miR128-2* and *miR421* expression transfected with miRNAs mimics in MCF-7 cells. **(B)** Cell cycle distribution of MCF-7 cells after overexpression of *miR128-1*, *miR128-2* and *miR421*. **(C)** Cell growth curve of MCF-7 cells with *miR128-1*, *miR128-2* and *miR421* overexpression. Data in **A** and **B** represent mean ± standard deviation (SD). Data were analyzed by unpaired two-tailed Student’s t-test (**A, B)** or **(C)** two-way ANOVA with Bonferroni correction. ns, no significant, * P < 0.05, **P < 0.01, ***P < 0.001.
